# Relationship between Statin Utilization and Socioeconomic Deprivation in Hungary

**DOI:** 10.3389/fphar.2016.00066

**Published:** 2016-03-24

**Authors:** Klára Boruzs, Attila Juhász, Csilla Nagy, Róza Ádány, Klára Bíró

**Affiliations:** ^1^Department of Health Systems Management and Quality Management in Health Care, Faculty of Public Health, University of DebrecenDebrecen, Hungary; ^2^Public Health Administration Service of Government Office of Capital City BudapestBudapest, Hungary; ^3^Department of Preventive Medicine, Faculty of Public Health, University of DebrecenDebrecen, Hungary; ^4^MTA-DE Public Health Research Group of the Hungarian Academy of Sciences, University of DebrecenDebrecen, Hungary

**Keywords:** statin, prescription, redemption, deprivation, cardiovascular mortality, health services research

## Abstract

The risk of premature mortality caused by cardiovascular diseases (CVDs) is approximately three times higher in the Central Eastern European region than in high income European countries, which suggests a lack and/or ineffectiveness of preventive interventions against CVDs. The aim of the present study was to provide data on the relationship between premature CVD mortality, statin utilization as a preventive medication and socioeconomic deprivation at the district level in Hungary. As a conceptually new approach, the prescription of statins, the prescription redemption and the ratio between redemption and prescription rates were also investigated. The number of prescriptions for statins and the number of redeemed statin prescriptions were obtained from the National Health Insurance Fund Administration of Hungary for each primary healthcare practice for the entire year of 2012. The data were aggregated at the district level. To define the frequency of prescription and of redemption, the denominator was the number of the 40+-year-old population adjusted by the rates of 60+-year-old population of the district. The standardized mortality rates, frequency of statin prescriptions, redeemed statin prescriptions, and ratios for compliance in relation to the national average were mapped using the “disease mapping” option, and their association with deprivation (tertile of deprivation index as a district-based categorical covariate) was defined using the risk analysis capabilities within the Rapid Inquiry Facility. The risk analysis showed a significant positive association between deprivation and the relative risk of premature cardiovascular mortality, and a reverse J-shaped association between the relative frequency of statin prescriptions and deprivation. Districts with the highest deprivation showed a low relative frequency of statin prescriptions; however, significantly higher primary compliance (redemption) was observed in districts with the highest deprivation. Our data clearly indicate that insufficient statin utilization is strongly linked to the so-called physician-factor, i.e., a statin prescription. Consequently, statin treatment is poor and represents a significant barrier to reducing mortality, particularly among people living in highly deprived areas of the country.

## Introduction

The health status of the population of Central Eastern European (CEE) member states of the European Union is less favorable than that of the countries that became members before May 2004 (EU15 countries). Although the period of epidemiological crisis plateaued between 1980 and the early 1990s in CEE countries and although the mortality caused by cardiovascular diseases (CVDs) is continuously decreasing, the relative risk of premature death, i.e., the ratio between the death rates for CEE countries compared with that of EU15 countries, is highly unfavorable. In the Visegrad Group countries (Czech Republic, Hungary, Poland, and Slovakia), the relative risk of early death caused by CVDs varies between 1.96 and 3.18 (Czech Republic, 1.96; Poland, 2.57; Slovakia, 2.91, and Hungary, 3.18) according to the latest available data (WHO HFA, 2015). These figures clearly indicate that the effectiveness of preventive interventions against CVDs is not sufficient in these countries; therefore, identifying gaps and improving the scale and effectiveness of preventive interventions are necessary in the region.

In addition to lifestyle modifying interventions, considerable benefit can be derived from preventive medication, including lipid-lowering therapy, particularly statin treatment (Perk et al., 2012; Kypridemos et al., 2015). A meta-analysis of 10 randomized trials enrolling a total of 70,388 people with a mean follow-up of 4.1 years clearly showed that statin treatment significantly reduced the risk of all-cause mortality (odds ratio 0.88), major coronary events (0.70), and major cerebrovascular events (0.81). In patients without established CVD but with cardiovascular risk factors, statin use was associated with significantly improved survival and large reductions in the risk of major cardiovascular events (Brugts et al., 2009). It was clearly demonstrated that every 1 mmol/l decrease in LDL cholesterol results in a 21% decrease in cardiovascular events (Yusuf et al., 2009). Therefore, statins are considered the first choice of drugs for patients with hypercholesterolemia or combined hyperlipidemia to reduce their risk of CVDs. Despite the presently growing number of publications on certain adverse effects of statin medication, it is considered to be obvious that the benefits of statins far outweigh the risks for the vast majority of patients (Godlee, 2014).

Although epidemiological data clearly demonstrate that cardiovascular mortality is much higher in CEE countries (WHO HFA, 2015), no studies on the relationship between statin utilization and socioeconomic characteristics of certain population groups were performed in these countries thus far. For the most developed countries, a large number of studies were published on the effect of socioeconomic status on statin utilization, with highly contradictory findings. Studies from Australia, Sweden, Denmark and the US (Stocks et al., 2004; Thomsen et al., 2005; Franks et al., 2010; Ohlsson et al., 2010) suggest that statin prescription in these countries has a socioeconomic gradient, typically among men, with women having a lower prevalence of statin use with increasing socioeconomic status. Furthermore, there is a less than expected utilization among the more disadvantaged. In a pharmacoepidemiological cohort study (Wallach-Kildemoes et al., 2012) it was clearly demonstrated that the Danish implementation of the high-risk strategy to prevent CVD by initiating statin therapy is inequitable across socioeconomic groups, reaching primarily high-risk individuals in groups with lower risk socioeconomic position. However, independent reports from the UK confirm that statin prescription is higher in more deprived areas (Ashworth et al., 2007; Wu et al., 2013). Similarly in New Zealand, those in the most deprived socioeconomic areas were most likely to receive statins. At ages of up to 75 years old, the use was higher among Maoris than non-Maori, particularly in middle age range (in the 45–54 age group, 11.6% of Maori received a statin prescription compared with 8.7% of non-Maoris) (Norris et al., 2014). In these studies, statin utilization was characterized by the frequency of prescription for statins, but no data were published on whether the patients redeemed their statin prescription (the rate of primary non-compliance). However, previous studies found that the overall adherence to treatment is low if statins are used for primary prevention, such as for patients with no previous cardiovascular events; with elevated cholesterol levels, only half of those patients prescribed a statin takes this medication on a regular basis (Mann et al., 2007). Long-term adherence to preventive statin therapy was also found to be decreased with decreasing income, especially in men aged 40–64 years in Denmark (Wallach-Kildemoes et al., 2013).

The aims of our present study were to provide data on statin prescription and its relation to the socioeconomic characteristics of various population groups in Hungary and, as a conceptually new approach on the redemption and relationship between redemption and prescription rates, to define the contribution of patient and/or physician factors to the inefficiency of statin utilization, if one exists, on CVD prevention. Because physicians in general practice are the key persons that initiate, coordinate, and provide long-term follow-up for CVD prevention (Mauskop and Borden, 2011), our study was performed as a cross-sectional analysis utilizing data on statin prescription and redemption rates from all general practices in Hungary. Furthermore, and the ratios of the statin prescription and redemption rates were analyzed as functions of deprivation in the areas served.

## Materials and Methods

This study focused on the comparative analysis of data for prescriptions by general practitioners (GPs) and redeemed prescriptions for statins in Hungary during 2012, the last year for which all data necessary for the analysis were available in validated databases in a district level study design. The ratios between the number of redeemed prescriptions and that of the prescriptions for statins were used to characterize the level of primary non-compliance. The associations between deprivation and premature mortality caused by diseases of the circulatory system (ICD-10: I00-I99), particularly ischemic heart disease (ICD-10: I25), as well as deprivation and statin utilization (prescription, redemption, and ratio) were also assessed.

### Data

Administratively, Hungary is divided into 19 counties as well as the capital Budapest; thus, it has 20 European regions at the third level of the Nomenclature of Territorial Units for Statistics (NUTS). The counties are further subdivided into 198 districts constituting Local Administrative Units 1 (LAU1), formerly known as NUTS level 4 of Hungary^1^.

For the year 2012, the mortality data were obtained from the Hungarian Central Statistical Office, whereas population data were obtained from the Central Office for Administrative and Electronic Public Services. Both mortality and population data for the districts were stratified by 5-year age bands and sex.

The number of prescriptions for statins and the number of redeemed statin prescriptions were obtained from the National Health Insurance Fund Administration of Hungary for each primary healthcare practice for the entire year of 2012. According to Hungarian regulations, GPs can prescribe only one type of medicine as a 1-month dose for one prescription for people who are taking long term medications. The data were aggregated at the district level. To define the frequency of prescription and that of redemption, the denominator was the size of the 40+-year-old population that was adjusted for by the rate of the 60+-year-old population of the district.

### Deprivation Index (DI) Calculation

Deprivation index was used to provide information about socio-economic deprivation at the district level compared with the national average for 2011, the year of the last census in the country. Socio-economic indicators for the DI were selected from available data stored at the Regional Informational System of the Ministry of Local Government and Regional Development. The data were originally obtained from the Hungarian Central Statistical Office (Census, 2011) and the Hungarian Tax and Financial Control Administration (2011).

The method to calculate DI values was described previously (Juhasz et al., 2010) and was successfully used in former studies designed to characterize the association between deprivation and mortality amenable to healthcare (Nagy et al., 2012) as well as between deprivation and premature mortality due to alcoholic liver disease (Nagy et al., 2014) in Hungary. Briefly, the DI is based on seven elementary socio-economic indicators, including income, level of education, rate of unemployment, rate of one-parent families, rate of large families, density of housing and car ownership. The variables were transformed using the natural log transformation and standardization (*z*-scores). The district-specific index is a weighted sum of the *z*-scores, with higher values representing greater deprivation. The weight of each variable was determined on the basis of the standardized scoring coefficients using a principal component analysis. The areas with positive index values are districts with a lower socio-economic status compared with the national average, and the converse is shown in districts with negative index values.

### Study at the District Level

In Hungary, the number of GP practices operating in 1 781 municipalities was 6 658 in 2012. The size of the practices, as the number of clients served, varied widely (800–3000 persons/practice), and the average size was 1488 persons/practice. Generally, more family practices operate in higher populated municipalities, whereas one family practitioner serves more than one municipality in less populated areas. In addition, there are primary healthcare practitioners with obligations to provide in-area care and those without such obligations. Considering the facts, that the free choice of family physician is a norm in Hungary and the detailed population data for practices, as well as the statin utilization data by age group are not available due to personal privacy, and the DI is not available at the practice level to reduce the risk of misclassification, we aggregated all data to the district level. The deprivation for each district was calculated using the population weighted average of DI. All districts included in the analysis were classified into 3 groups or tertiles, ranging from the least deprived (tertile I) to the most deprived (tertile III), with each containing a third of the population.

Using the “disease mapping” option within the Rapid Inquiry Facility (RIF)(Beale et al., 2010), spatial patterns of cardiovascular mortality (ICD-10: I00-I99) and mortality due to chronic ischemic heart disease (ICD-10: I25) for the 40+ age group for 2012 were investigated and visualized at the district level. Indirectly, standardized mortality ratios were calculated using sex- and age-specific death rates for the Hungarian population. The frequency of prescriptions for statins, redeemed statin prescriptions, and the ratios for compliance in relation to the national average were also mapped using the RIF and their association with deprivation was defined using tertiles of DI as a district-based categorical covariate and the risk analysis capabilities of the RIF. Chi-square tests for homogeneity and linear trend were also performed to test the global association of DI and mortality as well as statin utilization.

## Results

### Association between Deprivation and Premature Mortality Caused by CVDs

**FIGURE 1:**
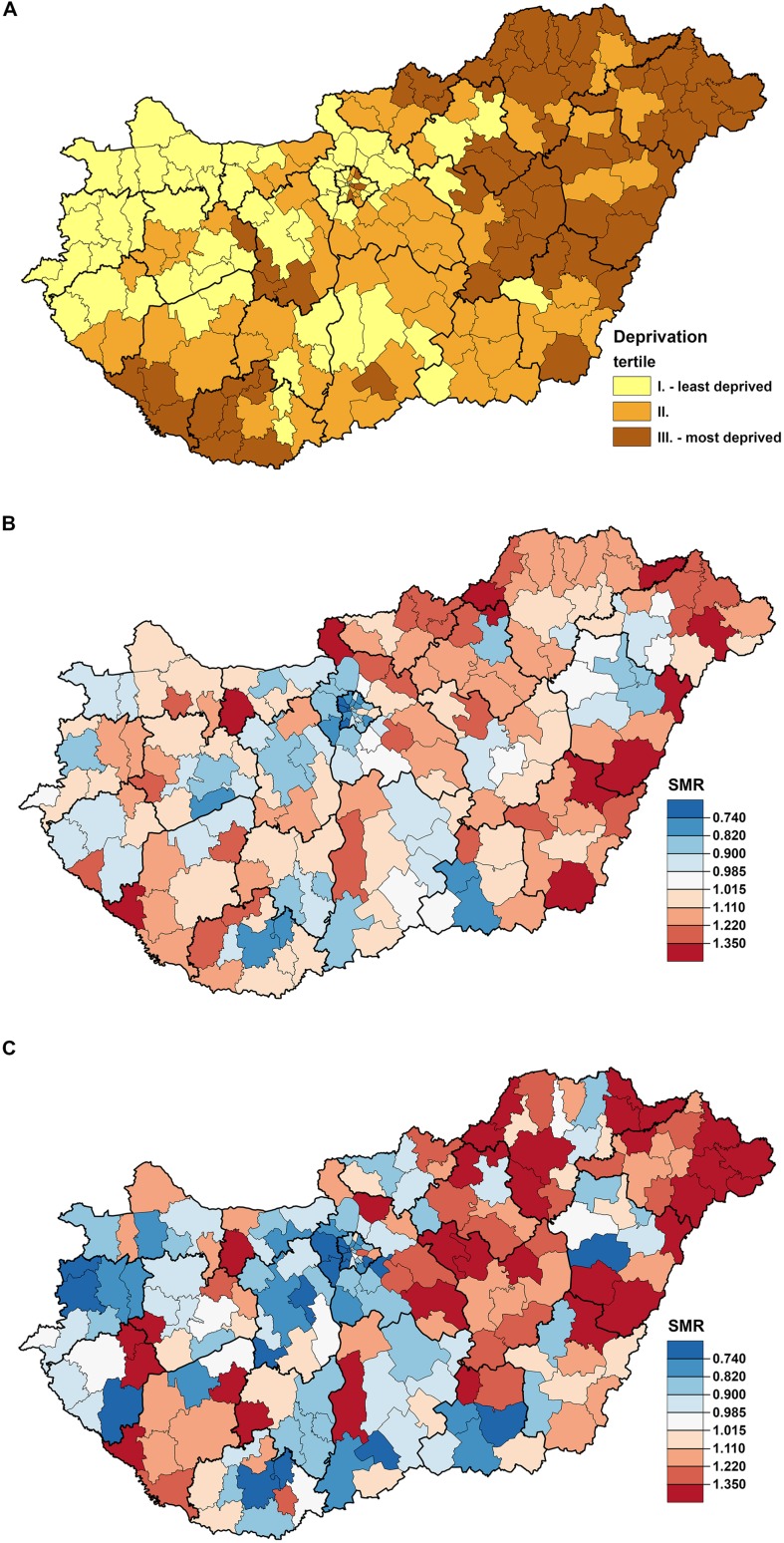
**The spatial distribution of deprivation (A) and premature mortality due to diseases of the circulatory system (ICD-10.: I00-I99) (B) as well as premature mortality due to chronic ischemic heart disease (ICD-10.: I25) (C) at the district level in Hungary, 2012**.

**FIGURE 2:**
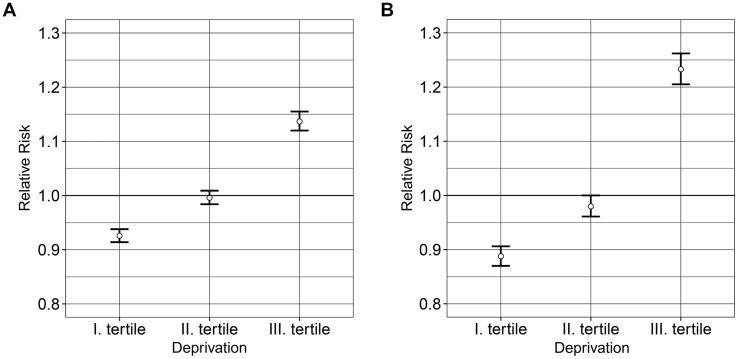
**Relationship between the deprivation tertiles and the relative risk of premature mortalities caused by diseases of the circulatory system (A) and chronic ischemic heart disease (B) for 2012 in Hungary**.

Deprivation index values defined by districts varied widely from –3.76 to +5.83, which indicates a high level of socio-economic inequalities in the country. The tertiles based on the DI values were defined as ranges of –3.76 ≤ DI ≤ –0.6, with an average of –1.32 (tertile I); –0.6 < DI ≤ 0.58, with an average of –0.04 (tertile II); and 0.58 < DI ≤ 5.83, with an average of 1.59. The least-favored districts were found in the northeastern and southwestern parts of Hungary in 2011, whereas the least deprived districts were located in the northwestern part of the country and in the capital city of Budapest and neighboring areas (**Figure 1A**). The spatial distribution of premature mortality due to diseases of the circulatory system (**Figure 1B**) and chronic ischemic heart disease (**Figure 1C**) in Hungary was also characterized by significant inequalities. The districts with the highest estimated SMRs are localized in the more deprived areas, particularly for chronic ischemic heart disease (**Figures 1A–C**; **Table 1**). This is supported by the results of the risk analysis showing a significant association between the relative risk of premature cardiovascular mortality and deprivation (χ^2^ homogeneity = 405.15, *P* = 0, χ^2^ linearity = 391.57, *P* = 0) as well as between deprivation and premature mortality caused by chronic ischemic heart disease (χ^2^ homogeneity = 443.89, *P* = 0, χ^2^ linearity = 412.96, *P* = 0) (**Figures 2A,B**).

**Table 1:** Mortality due to diseases of the circulatory system and chronic ischemic heart disease at district level by DI tertiles, Hungary, 2012.

DI tertiles	Mortality due to diseases of the circulatory system (ICD-10.: I00-I99)	Mortality due to chronic ischemic heart disease (ICD-10.: I25)
	Relative risk (95% CI)	Relative risk (95% CI)
I (Least deprived)	0.926 [0.914–0.938]	0.888 [0.870–0.906]
II	0.996 [0.984–1.009]	0.980 [0.961–1.000]
III (Most deprived)	1.137 [1.120–1.155]	1.233 [1.205–1.262]

### Statin Utilization (Prescription, Redemption, and Their Ratios)

In Hungary, a total of 10 044 005 statin prescriptions (simvastatin, atorvastatin, rosuvastatin, pravastatin, and fluvastatin) were prescribed in 2012, and only 63.39 [63.37–63.43] % (6 367 738) were redeemed. The frequency of prescription was 1.971 [1.9701–1.9725] and the frequency of redemption was 1.249 [1.248–1.251] per person aged 40+ years. These values were considered the national average.

**FIGURE 3:**
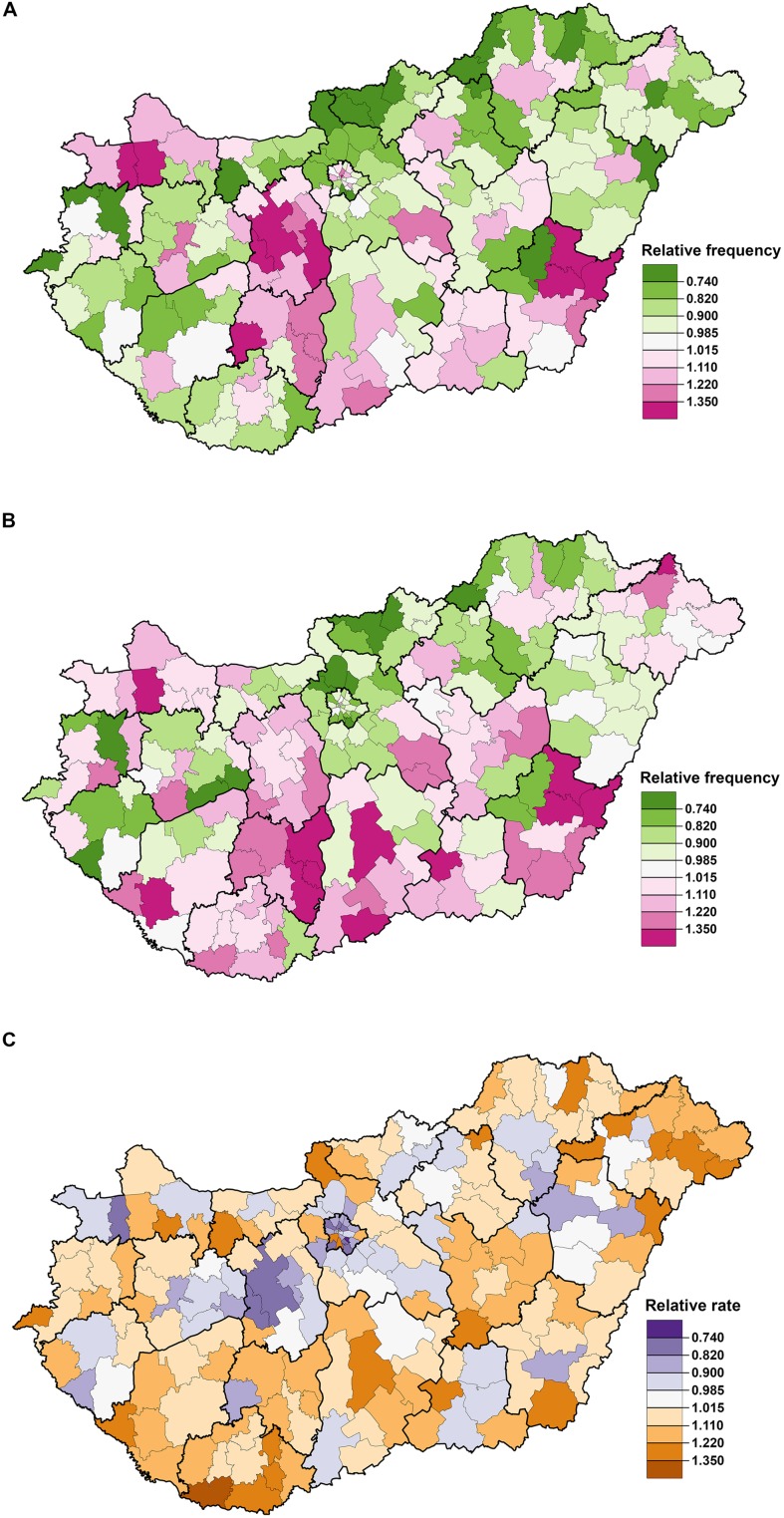
**The spatial distribution of the relative frequencies of statin prescription (A), redemption (B) and the relative redemption rate (relative compliance) (C) at the district level in Hungary, 2012**.

The frequency of statin prescriptions in relation to the national average was higher in districts in the northwestern and southeastern parts of Hungary and in the middle of the country (**Figure 3A**). The districts with a higher relative frequency of statin redemption were primarily located in the southern part of Hungary (**Figure 3B**). Districts with a higher redemption (primary compliance) rate were located along the axis in the northeastern-to-southwestern direction of Hungary, although the spatial distribution does not show clustering (**Figure 3C**).

**FIGURE 4:**
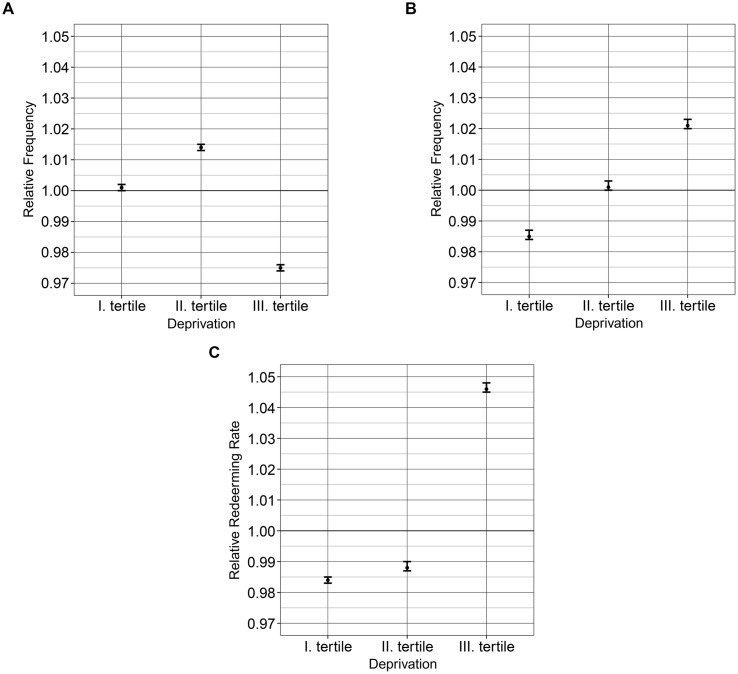
**Relationship between the deprivation tertiles and the relative frequencies of statin prescription (A), redemption (B) as well as the relative redemption rate (C) for 2012 in Hungary**.

The results of the risk analysis showed a reverse J-shaped association between the relative frequency of statin prescriptions and deprivation (**Figure 4A**). The areas of highest deprivation showed a low relative frequency of statin prescriptions (1.89 per person). A positive association was observed for the frequency of statin redemption by degree of deprivation (**Figure 4B**). Significantly higher compliance was observed in districts with the highest deprivation (67.88% [67.812–67.951]) (**Figure 4C**; **Table 2**).

**Table 2:** Relative frequencies of prescription of statins, statin redeeming, and relative redeeming rates at district level by DI tertiles, Hungary, 2012.

DI tertiles	Prescription of statins	Statin redeeming	Relative compliance
	Relative frequency (95% CI)	Relative frequency(95% CI)	Relative redeeming rate (95% CI)
I (Least deprived)	1.001 [1.000–1.002]	0.985 [0.984–0.987]	0.984 [0.983–0.985]
II	1.014 [1.013–1.015]	1.001 [1.000–1.003]	0.988 [0.987–0.990]
III (Most deprived)	0.975 [0.974–0.976]	1.021 [1.020–1.023]	1.046 [1.045–1.048]

## Discussion

Our present study was designed to answer the question whether a lack and/or ineffectiveness of preventive medication may also exist behind the about three times higher risk of premature mortality caused by cardiovascular diseases in the Central Eastern European region than in high income European countries; hypothesising that preventive medication is not adequately reaching people characterized by having higher risk of CVD, especially in groups living in socioeconomically deprived conditions. Analysing data on the relationship between premature CVD mortality, statin utilization (the prescription of statins, the prescription redemption and the ratio between redemption and prescription rates) as a preventive medication and socioeconomic deprivation at the district level in Hungary, a significant positive association between deprivation and the relative risk of premature cardiovascular mortality, and a reverse J-shaped association between the relative frequency of statin prescriptions and deprivation were found. In districts with the highest deprivation a low relative frequency of statin prescriptions was detected; however, significantly higher primary compliance (redemption) was observed in these districts.

The 2012 guidelines from the Fifth Joint Task Force of the European Societies on CVD Prevention in Clinical Practice recommend that all hypertensive patients with established CVD or with type 2 diabetes and patients with an estimated 10-year risk of cardiovascular death ≥5% based on the SCORE chart should be considered for statin therapy (Stone et al., 2014). The 2013 AHA/ACC (American College of Cardiology Foundation/American Heart Association) guidelines on the management of elevated blood cholesterol for the primary and secondary prevention of atherosclerotic CVDs recommend appropriate levels of statin therapy for different risk groups (high intensity in patients with atherosclerotic CVD, moderate or high intensity in patients with diabetes depending on their 10-year risk of atherosclerotic CVD, and moderate or high intensity in individuals aged 40 to 75 years without CVD or diabetes but with a 10-year risk of clinical events >7.5% and an LDL-cholesterol level of 1.8–4.9 mmol/l) (Yancy et al., 2013). The WHO guidelines for the prevention of CVDs propose preventive medication to the lower cholesterol level in all individuals with total cholesterol at or above 8 mmol/l, but if the ten-year CVD risk is 20% or higher, adults aged >40 years with a cholesterol level >5.0 mmol/l and/or LDL cholesterol > 3.0 mmol/l despite a lipid-lowering diet should be given a statin.

It is generally accepted, with minor limitations, that for dyslipidemic persons who have not already had a vascular event but are at a higher cardiovascular risk, combined statin therapy substantially reduces the CVD mortality risk, thereby “potentially being an ideal risk-reducing factor with added risk reduction by lifestyle changes” (Opie, 2015). The evidence for statins for secondary prevention in patients after a heart attack is more robust, decreasing the risk of a second heart attack by approximately one-third (Wei et al., 2005). Because in CEE countries, including Hungary, the premature mortality caused by CVDs is significantly higher than in the most developed countries of the European Region, it is reasonable to suggest that preventive interventions, such as preventive medication with statins, have not been considered or implemented sufficiently.

Our results are consistent with findings published for different countries (Mackenbach et al., 2000, 2003) and clearly show that standardized mortality rates caused by CVDs, particularly from chronic ischemic heart disease, are significantly higher in districts with the highest DIs (grouped into tertile III) in Hungary. Although premature mortality caused by CVDs, particularly by ischemic heart disease, is highest in the same districts, the frequency of statin prescriptions at the primary care level was significantly lower than the national average. However, the rate of redemption, and consequently, the ratio between redemption and prescription rates were significantly higher. These data clearly indicate that insufficient statin utilization is highly linked to the so-called physician-factor; i.e., statin prescription, consequently statin treatment is poor and represents significant barriers to mortality reductions, particularly among people living in highly deprived areas of the country. Because the socio-economic gap in health and mortality is widening in Europe – as the largest study to have explored the association between social factors and serious outcomes of chronic diseases, particularly cardiovascular outcomes clearly demonstrated (Mackenbach et al., 2003) – the identification of gaps in preventive services in deprived areas is of the utmost importance. The statement “reducing socio-economic inequalities in mortality in Western Europe critically depends upon speeding up mortality declines from CVDs in lower socioeconomic groups, and countering mortality increases from several other causes of death in lower socioeconomic groups” can be interpreted as an imperative on how to improve the health of the population for the low- and middle-income countries of the European Region. Our study identifies a gap in current cardiovascular prevention practice by showing that many patients are likely under-treated and others remain untreated.

Regarding the reasons and remedies for under-treatment, it seems likely that the lack of financial incentives for primary prevention at the level of primary care (Adany et al., 2013) has a strong effect, particularly if it meets the low health literacy of people living in the most deprived districts. Although a survey (UNDP/World Bank/EC regional Roma survey 2011) revealed a high percentage of households unable to afford prescription medication in eleven countries of the CEE region, including Hungary, our result showing significantly higher redemption rates in the most deprived districts of the country indicates that for statin prescription, the primary non-compliance, for any reason, is not a factor highly defining the insufficient preventive medication. Based on our results, the importance of determining why GPs do not follow guideline recommendations regarding lipid-lowering treatment should be emphasized. Benefits provided by the National Health Insurance Fund of Hungary include cost-free healthcare services, such as preventive examinations, primary healthcare, and drug reimbursement on grounds of equity. For persons who are low income or on social welfare, medical exemption certificates are available, such that they are exempt from prescription charges in the case of defined medicaments, including the majority of statin medicines.

The limitations of our study deserve mention. All three factors (patient, physician, and health system) that have an effect on statin utilization cannot be covered in a single study. The effects associated with socio-economic factors may be mediated by other factors that were not included in our analyses. Health system factors were only partially studied, and access to care requires particular attention in further studies. Information about various elements that may influence a patient’s likelihood to take statin medications should also be collected to understand the relatively low redemption rate in areas with the lowest deprivation indices.

## Author Contributions

The study was designed and the manuscript was written by RÁ. KB, AJ, CN, and KBíró have contributed to the analysis of the data. All authors stated above have contributed to the interpretation of the results, and helped to draft the manuscript.

## Conflict of Interest Statement

The authors declare that the research was conducted in the absence of any commercial or financial relationships that could be construed as a potential conflict of interest.

## Footnotes

1 http://ec.europa.eu/eurostat/ramon/statmanuals/files/KS-RA-07-005-EN.pdf
